# *Schleiferilactobacillus harbinensis JNDM* Postbiotics Alleviate Atopic Dermatitis with Concurrent Changes in Gut Microbiota and Fecal SCFAs

**DOI:** 10.3390/microorganisms14040913

**Published:** 2026-04-17

**Authors:** Zhijie Shi, Ke Li, Jiaqian Liang, Laifa Yan, Yuzhen Guo, Zhenming Lu, Xiaojuan Zhang, Hongyu Xu, Jinsong Shi

**Affiliations:** 1 School of Life Science and Health Engineering, Jiangnan University, Wuxi 214122, China; 6233304029@stu.jiangnan.edu.cn; 2 Key Laboratory of Industrial Biotechnology of Ministry of Education, School of Biotechnology, Jiangnan University, Wuxi 214122, China; like9801@163.com (K.L.); 6230209014@stu.jiangnan.edu.cn (J.L.); 6240210100@stu.jiangnan.edu.cn (L.Y.); 6240210011@stu.jiangnan.edu.cn (Y.G.); zmlu@jiangnan.edu.cn (Z.L.); zhangxj@jiangnan.edu.cn (X.Z.)

**Keywords:** *Schleiferilactobacillus harbinensis*, postbiotics, atopic dermatitis, gut–skin axis, *Alistipes*, butyrate

## Abstract

Atopic dermatitis (AD) is a chronic inflammatory dermatosis driven by skin barrier dysfunction, immune dysregulation, and gut–skin axis imbalance. While probiotics show promise, the therapeutic potential and mechanisms of topical postbiotics in modulating the gut–skin axis remain understudied. Here, we investigated the efficacy of *Schleiferilactobacillus harbinensis JNDM*-derived cell-free supernatant (CFS) and lysate (ShL) in a DNFB-induced AD mouse model. Topical application of both CFS and ShL significantly attenuated AD-like symptoms, reduced epidermal thickening, and restored the expression of the barrier protein filaggrin. Immunologically, treatment suppressed the Th2-dominant inflammatory cascade (IL-4, IL-5, IL-13, IL-33, TSLP) and reduced serum IgE and IFN-γ levels. Notably, ShL exhibited superior systemic efficacy, significantly inhibiting mast cell infiltration and reducing the spleen index. 16S rRNA sequencing revealed that topical intervention remotely remodeled the gut microbiota, specifically reversing the depletion of the beneficial genus *Alistipes* and suppressing the compensatory increase in *Odoribacter*. This microbial restructuring was accompanied by distinct metabolic changes: ShL treatment resulted in an approximately 4-fold elevation in fecal butyrate concentrations compared with the model group. Correlation analysis further validated a strong positive axis linking *Alistipes* abundance and butyrate levels to skin barrier integrity. Collectively, our findings demonstrate that *S. harbinensis* postbiotics—particularly the lysate—ameliorate AD through a dual mechanism of local barrier repair and systemic metabolic modulation via the gut–skin axis, presenting a promising non-steroidal therapeutic strategy.

## 1. Introduction

Atopic dermatitis (AD) is a prevalent, chronic, and relapsing inflammatory skin disorder that imposes a significant global health burden, affecting 15–20% of children and 1–3% of adults worldwide [1,2,3]. The clinical manifestation of AD is characterized by intense pruritus, xerosis, and eczematous lesions, often serving as the initial step in the “atopic march,” which predisposes individuals to subsequent allergic conditions such as asthma and allergic rhinitis [4]. The etiology of AD is multifactorial, involving a complex interplay between epidermal barrier dysfunction, innate and adaptive immune dysregulation (typically a Th2/Th1 imbalance), and environmental triggers [5]. Current pharmacological strategies, including topical corticosteroids and calcineurin inhibitors, are the standard of care but are frequently associated with adverse effects, such as cutaneous atrophy and systemic immunosuppression, particularly with long-term use in pediatric populations [6]. Consequently, there is an urgent need for safer, efficacious therapeutic alternatives derived from natural sources.

Emerging research has illuminated a critical bidirectional communication network known as the “gut-skin axis,” which links the immunological and metabolic environments of the intestine and the skin [7,8,9,10,11,12]. Although anatomically distinct, both organs serve as primary immune barriers heavily colonized by microbiota. Dysbiosis in the gut microbiota is increasingly recognized as a driving factor in AD pathogenesis, as it disrupts the production of immunomodulatory metabolites—most notably short-chain fatty acids (SCFAs) like butyrate and propionate [13]. These metabolites are pivotal in expanding regulatory T cells (Tregs) and maintaining systemic immune homeostasis. Therefore, therapeutic strategies that can restore gut microbial balance and SCFA production offer a promising avenue for managing cutaneous inflammation [14].

Probiotics have demonstrated potential in modulating the gut—skin axis; however, the application of live bacteria to compromised skin poses risks of infection, particularly in immunocompromised AD patients. This has shifted scientific interest toward “postbiotics”—non-viable bacterial products or metabolic byproducts (such as cell-free supernatants and lysates) [15]. Postbiotics retain the beneficial immunomodulatory and barrier-repairing properties of probiotics while offering superior safety, stability, and ease of formulation [16]. While the oral administration of probiotics is well-studied, the potential of topical postbiotic application to exert systemic effects via the gut—skin axis remains an underexplored but highly significant area of research.

*Schleiferilactobacillus harbinensis JNDM* is a lactic acid bacterium originally isolated from traditional fermented vegetables. Genomic and phenotypic characterizations have confirmed its safety and demonstrated an apparently robust capacity to produce bioactive exopolysaccharides (EPSs) that promote the growth of beneficial gut bacteria and enhance SCFA production [17,18]. Despite these promising attributes, the therapeutic potential of *S. harbinensis*-derived postbiotics in AD, particularly regarding their ability to modulate the gut–skin axis via topical application, has not been investigated. Furthermore, it remains unclear whether the therapeutic efficacy differs between its secreted metabolites (Cell-Free Supernatant, CFS) and its intracellular/cell-wall components (Lysate, ShL).

Therefore, the aim of this study was to investigate the anti-atopic dermatitis potential of *Schleiferilactobacillus harbinensis JNDM*-derived postbiotics and to explore their possible association with gut microbiota and SCFA alterations. To address these objectives, ShP was first used in vitro as a crude whole-postbiotic preparation for preliminary bioactivity screening, whereas the culture was subsequently fractionated into CFS and ShL for in vivo comparative evaluation of extracellular soluble and cell-associated activities. By integrating cellular assays, a DNFB-induced AD mouse model, and gut microbiota- and SCFA-related analyses, this study aimed to provide a more comprehensive assessment of the therapeutic potential of *S. harbinensis* JNDM-derived postbiotics.

## 2. Materials and Methods

### 2.1. Preparation of Schleiferilactobacillus harbinensis JNDM Components

*Schleiferilactobacillus harbinensis JNDM* was obtained from the System Fermentation Laboratory, Jiangnan University, and cultured in MRS broth (Qingdao Haibo Biotechnology Co., Ltd., Qingdao, China) at 37 °C for 24 h to stationary phase (OD_600_ ≈ 5.0).

For in vitro experiments, a crude whole-postbiotic preparation (ShP) was generated by disrupting the culture using a high-pressure homogenizer (AH-1500, Antuos Nanotechnology (Suzhou) Co., Ltd., Suzhou, China) at 1000 bar for three cycles.

For in vivo experiments, the culture was centrifuged at 12,000× *g* for 5 min at 4 °C. The supernatant was collected and filtered through a 0.22 μm membrane to obtain the cell-free supernatant (CFS). The bacterial pellet was washed twice with sterile PBS, resuspended to the original volume, and disrupted under the same high-pressure homogenization conditions (1000 bar, three cycles) to obtain the bacterial lysate (ShL). ShP was used for in vitro screening, while CFS and ShL were used for in vivo experiments.

### 2.2. Cell Culture and Wound Healing Assay

HaCaT cells were maintained in DMEM (QIDU BIOPHARMACEUTICAL, Zibo, China) supplemented with Fetal Bovine Serum (Prime, FSP500, Excell, Guangzhou, China) and 1% penicillin–streptomycin (Shanghai dowobio Biotechnology Co., Ltd., Shanghai, China) at 37 °C in a humidified 5% CO_2_ atmosphere. For the wound healing assay, cells were seeded and cultured for 24 h. Atopic-like inflammation was induced using 10 ng/mL TNF-α (Solarbio, Beijing, China) and IFN-γ (Solarbio, Beijing, China). A scratch wound was created, and detached cells were removed by rinsing. Cells were then treated with varying concentrations of ShP. Wound closure was monitored microscopically at 0 and 24 h. The cell pellets were collected for RT-qPCR analysis of inflammatory markers. Gene expression levels were normalized to the housekeeping gene GAPDH. Relative gene expression was calculated using the 2^−ΔΔCt^ method. All reactions were performed in technical triplicate. Amplification specificity was verified by melt curve analysis. Primer sequences used in this study are listed in Table 1.

### 2.3. Anti-Inflammatory Assay in RAW264.7 Cells

RAW264.7 macrophages were cultured in DMEM with 10% FBS and 1% penicillin–streptomycin at 37 °C in 5% CO_2_. Cells were incubated for 16 h prior to experimentation. Inflammation was induced using 1 μg/mL LPS (Solarbio, Beijing, China), followed by treatment with different concentrations of ShP for 24 h. Nitric oxide (NO) production was assessed by measuring nitrite levels in the culture supernatant. The cell pellets were collected for RT-qPCR analysis of inflammatory markers. Gene expression levels were normalized to the housekeeping gene GAPDH. Relative gene expression was calculated using the 2^−ΔΔCt^ method. All reactions were performed in technical triplicate. Amplification specificity was verified by melt curve analysis. Primer sequences used in this study are listed in Table 2.

### 2.4. Animal Experimental Design

Specific-pathogen-free (SPF) male BALB/c mice (6–8 weeks old, 25–30 g) were obtained from GemPharmatech Co., Ltd. (Nanjing, Jiangsu, China) and housed under controlled conditions (25 ± 2 °C, 50% humidity, 12 h light/dark cycle) with free access to food and water. All procedures were approved by the Ethics Committee of Jiangnan University (JN. No20240530b0420820[255]). Mice were acclimatized for one week prior to experimentation. Each mouse was considered an experimental unit. This animal study is reported in accordance with the ARRIVE 2.0 guidelines, and a completed ARRIVE checklist is provided as Supplementary Material.

Animals were randomly assigned to five groups (*n* = 6 per group): Normal Control (NC), Model (M), Positive Control (CRI), Cell-Free Supernatant (CFS), and Lysate (ShL). Randomization was performed using a computer-generated random number table (SPSS 26.0). Sample size was determined based on preliminary experimental observations to ensure adequate statistical power for detecting differences in dermatitis score.

AD-like dermatitis was induced using 2,4-dinitrofluorobenzene (DNFB) [19]. After shaving the dorsal skin, mice were sensitized with 0.5% DNFB (acetone/olive oil, 4:1 *v*/*v*) on days 2 and 5. From day 10, mice were challenged with 0.2% DNFB every other day for a total of six applications (days 10–20). Treatments were applied topically every other day during the challenge period. NC and M groups received normal saline (50 μL/cm^2^), the CRI group received crisaborole ointment (15 mg/cm^2^), the CFS group received cell-free supernatant (50 μL/cm^2^), and the ShL group received bacterial lysate (200 μL per mouse).

Animals were monitored daily for health status. Predefined exclusion criteria included body weight loss exceeding 20% or severe skin damage unrelated to DNFB treatment; no animals met the exclusion criteria. On day 21, mice were euthanized under isoflurane anesthesia after 12 h fasting. Skin, serum, spleen, and fecal samples were collected for analysis. The primary outcome measure used to evaluate treatment efficacy was dermatitis score, which guided the sample size selection. Secondary outcome measures included epidermal thickness, cytokine expression, immune cell distribution, SCFA concentrations, and microbiota composition. Outcome assessment was performed using coded group labels to minimize observer bias where applicable.

### 2.5. Evaluation of Dermatitis Scores

Dermatitis severity was evaluated in a blinded manner using coded group identifiers, based on erythema/hemorrhage, dryness, erosion, and excoriation (0–4 per item). The total score was the sum of the four items. Histological scoring and downstream data analyses were also performed using blinded sample labels where applicable.

### 2.6. Histological Analysis

Skin tissues were fixed in 4% paraformaldehyde, paraffin-embedded, and sectioned at 5 μm thickness. H&E staining was used to assess histopathology and epidermal thickness, and toluidine blue staining was used for mast cell visualization. For quantitative analysis, three sections per mouse and three randomly selected microscopic fields per section were analyzed at 200× magnification. Epidermal thickness was measured using ImageJ 1.54i software. Inflammatory cell infiltration was evaluated using a semi-quantitative scoring scale (0–4), and mast cells were counted in toluidine blue-stained dermal regions.

All analyses were performed using coded sample identifiers to ensure blinded evaluation.

### 2.7. Quantitative Real-Time Polymerase Chain Reaction (RT-qPCR) Analysis

Total RNA was extracted from dorsal skin tissues using TRIzol reagent (abm, Vancouver, WA, Canada) and homogenized using a mini bead beater (abm, Vancouver, WA, Canada). Following RNA isolation, 1000 ng of total RNA was reverse-transcribed into cDNA using the 5× All-In-One RT MasterMix (abm, Vancouver, WA, Canada) according to the manufacturer’s instructions. RT-qPCR was subsequently performed on a Bio-Rad CFX96 Real-Time Quantitative PCR System (Hercules, CA, USA) using BlastTaq™ 2× qPCR MasterMix (abm, Vancouver, WA, Canada). Gene expression levels were normalized to the housekeeping gene GAPDH. Relative gene expression was calculated using the 2^−ΔΔCt^ method. All reactions were performed in technical triplicate. Amplification specificity was verified by melt curve analysis. Primer sequences used in this study are listed in Table 3.

### 2.8. Immunological Markers

Serum levels of immunoglobulin E (IgE) and interferon-gamma (IFN-γ) were quantified using a Mouse IgE ELISA Kit (Elabscience, Wuhan, China) and a Mouse IFN-γ ELISA Kit (Nanjing Jiancheng Bioengineering Institute, Nanjing, China), respectively, following the manufacturers’ protocols.

### 2.9. Flow Cytometry Analysis of Splenic Lymphocytes

Spleens were mechanically dissociated in RPMI 1640 medium (QIDU Biopharmaceutical, Zibo, China) to obtain single-cell suspensions. Red blood cells were removed using lysis buffer (Elabscience, Wuhan, China), and cells were filtered through a 200-mesh nylon strainer.

For immunophenotyping, 1 × 10^6^ cells were stained with a Mouse Lymphocyte Subset Antibody Cocktail (Elabscience, Wuhan, China) including FITC anti-CD3 (Clone 17A2), Elab Fluor^®^ Violet 450 anti-CD4 (Clone GK1.5), Elab Fluor^®^ Violet 500 anti-CD8a (Clone 53-6.7), and PE/Cyanine7 anti-CD45R/B220 (Clone RA3-3A1/6.1) at 4 °C for 30 min in the dark. Compensation controls included single-stained and unstained samples.

Data were acquired on a BD FACS Aria III flow cytometer (BD Biosciences, San Diego, CA, USA), with at least 10,000 lymphocyte events collected per sample. Sequential gating included debris exclusion (FSC-A vs. SSC-A), doublet discrimination (FSC-H vs. FSC-A), identification of lymphocytes based on FSC-A versus SSC-A characteristics, and classification of CD3^+^ T cells and CD3-B220^+^ B cells. CD3^+^ T cells were further subdivided into CD4^+^ helper and CD8^+^ cytotoxic subsets. Representative gating plots are shown in Supplementary Figure S2.

### 2.10. Short-Chain Fat Acids (SCFAs) Metabolism

Fecal samples (0.1 g) were homogenized in 0.9 mL ultrapure water and centrifuged at 8000 rpm for 5 min. The supernatant was deproteinized using potassium ferricyanide and zinc sulfate solutions, centrifuged at 12,000 rpm for 2 min, and filtered through a 0.22 μm membrane. Concentrations of acetic, propionic, and butyric acids were determined by high-performance liquid chromatography (HPLC, Waters e2695, Cranbury, NJ, USA) equipped with a Carbomix H column (4.6 mm × 300 mm, 5 μm, Sepax, Suzhou, China). The mobile phase was 2.5 mM sulfuric acid at a flow rate of 0.6 mL/min, with a column temperature of 55 °C. Detection was performed using a PDA detector at 214 nm.

### 2.11. 16S rRNA Amplification Sequencing of Gut Microbiota

Microbial genomic DNA was extracted from fecal samples using the E.Z.N.A.^®^ Stool DNA Kit (Omega Bio-tek, Norcross, GA, USA) according to the manufacturer’s instructions. The V4–V5 region of the bacterial 16S rRNA gene was amplified using barcoded primers 515F and 907R and sequenced on an Illumina MiSeq platform (PE300 mode) (Shanghai BIOZERON Biotech Co., Ltd., Shanghai, China).

Raw reads were demultiplexed and quality-filtered using Trimmomatic (average quality score > 20; minimum length 50 bp). High-quality sequences were clustered into operational taxonomic units (OTUs) at 97% similarity using UPARSE, and chimeric sequences were removed using UCHIME. Taxonomic classification was performed using the SILVA reference database (SSU138.2).

Alpha diversity was evaluated using the Chao1 richness index and Faith’s phylogenetic diversity (Faith’s PD). Sequencing depth was normalized by rarefaction prior to diversity analysis to minimize bias caused by uneven sampling effort. Phylogenetic trees required for Faith’s PD calculation were generated based on aligned 16S rRNA sequences using FastTree.

Beta diversity was assessed using principal coordinate analysis (PCoA) based on the Bray–Curtis distance. Statistical significance of beta diversity differences among groups was assessed using permutational multivariate analysis of variance (PERMANOVA) based on the Bray–Curtis distance with 999 permutations using the vegan package in R.

Relative abundance of microbial taxa at the phylum and genus levels was calculated to evaluate compositional differences among groups. For differential abundance analysis, taxonomic abundance tables were first converted to relative abundance format. Low-abundance taxa were filtered prior to statistical testing to reduce noise. Differentially abundant taxa were then identified using LEfSe with an LDA score threshold > 2.0 and *p* ≤ 0.05. As a complementary sensitivity analysis, differential abundance was also assessed using MaAsLin3 to evaluate the robustness of taxa identified by LEfSe.

### 2.12. Data Analysis

Statistical analyses were performed using GraphPad Prism 9 (GraphPad Software, Boston, MA, USA), SPSS 27.0 (IBM Corp., Armonk, NY, USA), and R software (version 4.2.0). Data are presented as mean ± standard deviation (SD). Differences between two groups were evaluated using two-tailed Student’s *t*-test, while comparisons among multiple groups were performed using one-way analysis of variance (ANOVA) followed by Tukey–Kramer post hoc tests. Normality was assessed using the Shapiro–Wilk test and homogeneity of variance was evaluated using Levene’s test prior to parametric analysis. When assumptions were not satisfied, non-parametric alternatives (Mann–Whitney U test or Kruskal–Wallis test) were applied.

Beta diversity differences were analyzed using permutational multivariate analysis of variance (PERMANOVA) based on the Bray–Curtis distance with 999 permutations in R. Pairwise PERMANOVA comparisons between the model group and each treatment group were additionally performed, and *p* values were adjusted using the Benjamini–Hochberg method. Spearman correlation analysis was used to evaluate associations between microbial taxa, SCFAs, immune parameters, and barrier-related markers.

To reduce the likelihood of false-positive findings due to multiple testing, *p* values from correlation analyses and pairwise PERMANOVA were adjusted using the Benjamini–Hochberg false discovery rate (FDR) method where appropriate. A *p* value < 0.05 or FDR-adjusted *p* value < 0.05 was considered statistically significant.

## 3. Results

### 3.1. ShP Enhances Wound Healing and Barrier Function While Reducing Inflammation in HaCaT Cells

**Figure 1:**
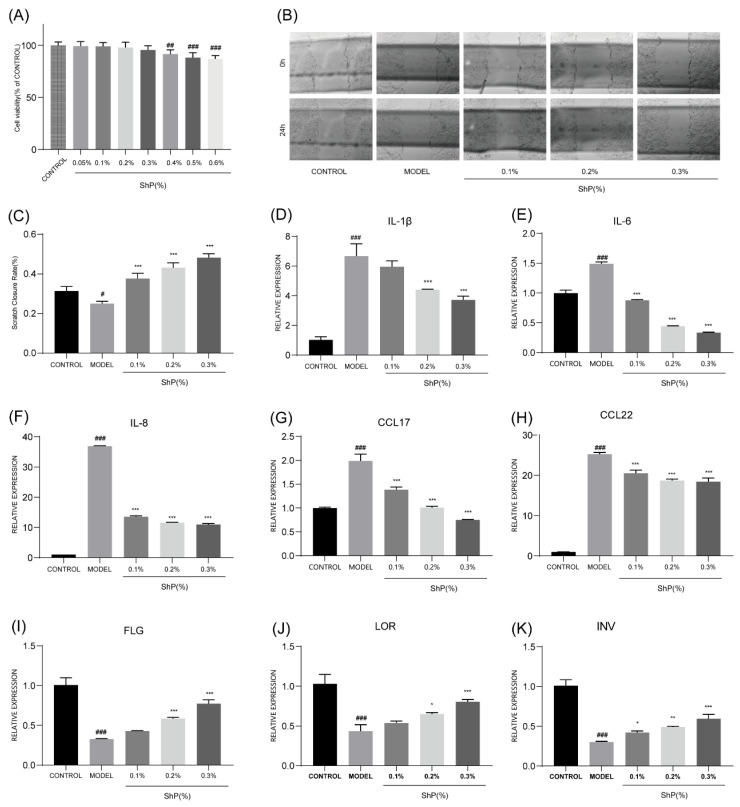
ShP promotes wound healing, suppresses inflammatory gene expression, and restores barrier-related gene expression in HaCaT cells. (**A**) Cell viability (% of CONTROL); (**B**) Wound healing assay images; (**C**) Scratch Closure Rate (%); (**D**) IL-1β; (**E**) IL-6; (**F**) IL-8; (**G**) CCL17; (**H**) CCL22; (**I**) FLG; (**J**) LOR; (**K**) INV. Data were expressed as mean ± SD (*n* = 3/group), * *p* < 0.05, ** *p* < 0.01, *** *p* < 0.001: compared with MODEL group; # *p* < 0.05, ## *p* < 0.01, ### *p* < 0.001: compared with CONTROL group.

To evaluate the regenerative potential of ShP, an in vitro scratch wound healing assay was performed. In the model group induced by TNF-α and IFN-γ, the migratory capacity of HaCaT cells was significantly impaired compared to the control group (*p* < 0.05). However, treatment with ShP (0.1%, 0.2%, and 0.3%) markedly promoted wound closure in a concentration-dependent manner without affecting cell viability (*p* < 0.001). Notably, the 0.3% ShP intervention exhibited the most potent effect (Figure 1A–C), suggesting that ShP effectively stimulates keratinocyte migration to accelerate tissue repair.

Concurrently, RT-qPCR analysis revealed that stimulation with TNF-α and IFN-γ triggered a relatively robust inflammatory response, characterized by the significant upregulation of pro-inflammatory cytokines (IL-1β, IL-6, IL-8) and chemokines (CCL17, CCL22) (*p* < 0.001). ShP treatment significantly attenuated the mRNA expression of these inflammatory mediators in a dose-dependent manner (Figure 1D–H). Furthermore, the expression of key skin barrier proteins—filaggrin (FLG), loricrin (LOR), and involucrin (INV)—was severely suppressed in the model group. ShP treatment effectively reversed this suppression, with the 0.3% concentration yielding a markedly significant upregulation of FLG, LOR, and INV (*p* < 0.001), suggesting that ShP contributes to the structural reconstruction of the epidermal barrier (Figure 1I–K).

### 3.2. ShP Inhibits Inflammatory Mediators in LPS-Stimulated RAW264.7 Macrophages

**Figure 2:**
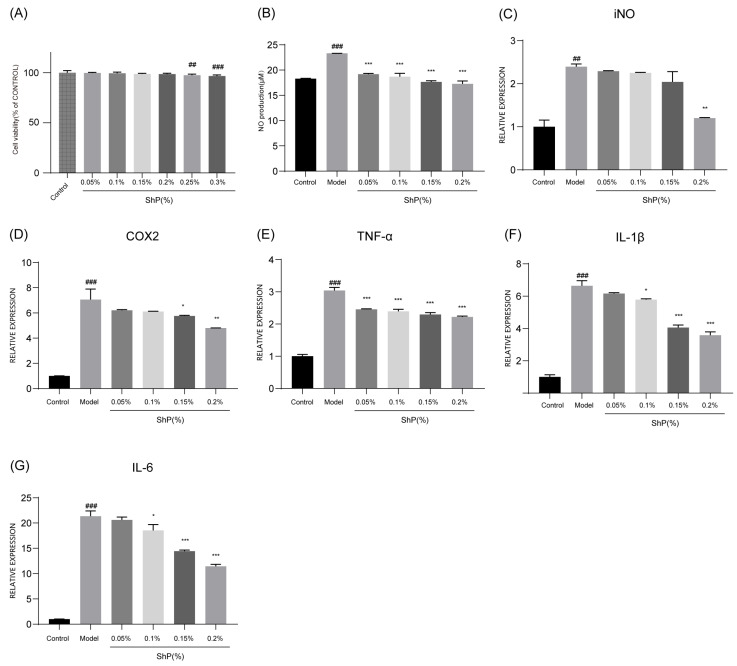
ShP suppresses LPS-induced inflammatory responses in RAW264.7 cells. (**A**) Cell viability after ShP treatment (normalized to the Control group). (**B**) Nitric oxide (NO) production in culture supernatants. (**C**,**D**) RT-qPCR analysis of iNOS and COX-2. (**E**–**G**) RT-qPCR analysis of pro-inflammatory cytokines: TNF-α, IL-1β, and IL-6. RAW264.7 cells were stimulated with LPS and treated with ShP at the indicated concentrations for 24 h. Data are presented as mean ± SD (n = 3/group). * *p* < 0.05, ** *p* < 0.01, *** *p* < 0.001: compared with MODEL group; ## *p* < 0.01, ### *p* < 0.001: compared with CONTROL group.

To further elucidate the anti-inflammatory mechanism, we examined the effects of ShP on LPS-stimulated RAW264.7 macrophages. Stimulation with LPS led to a surge in nitric oxide (NO) production (*p* < 0.001) and a corresponding upregulation of inducible nitric oxide synthase (iNOS) (*p* < 0.01) and cyclooxygenase-2 (COX2) mRNA expression (*p* < 0.001). ShP treatment (0.05%, 0.10%, 0.15% and 0.20%) significantly inhibited NO secretion and downregulated iNOS and COX-2 levels (Figure 2A–D), effectively blocking the synthesis of these key inflammatory mediators. Additionally, ShP treatment resulted in a marked, dose-dependent reduction in the mRNA expression of the pro-inflammatory cytokines TNF-α, IL-1β, and IL-6 (Figure 2E,F). These findings suggest that ShP exerts its anti-inflammatory effects by suppressing the transcriptional activation of inflammatory cascades in macrophages.

### 3.3. CFS and ShL Ameliorate Symptoms and Histopathological Changes in AD Mice

Collectively, the in vitro findings demonstrate that the crude postbiotic preparation exerts significant barrier-repairing and anti-inflammatory effects. However, ShP represents a complex mixture containing both extracellular metabolites and intracellular/cell-wall components. It remains unclear whether the observed therapeutic benefits are primarily attributable to secreted bioactive molecules or to structural cellular fractions. Moreover, confirming whether these cellular effects translate into in vivo therapeutic efficacy is essential. To address these questions, we fractionated the *S. harbinensis JNDM* culture into two distinct components—Cell-Free Supernatant (CFS) and *S. harbinensis JNDM* Lysate (ShL)—and comparatively evaluated their protective effects in a DNFB-induced AD mouse model.

**Figure 3:**
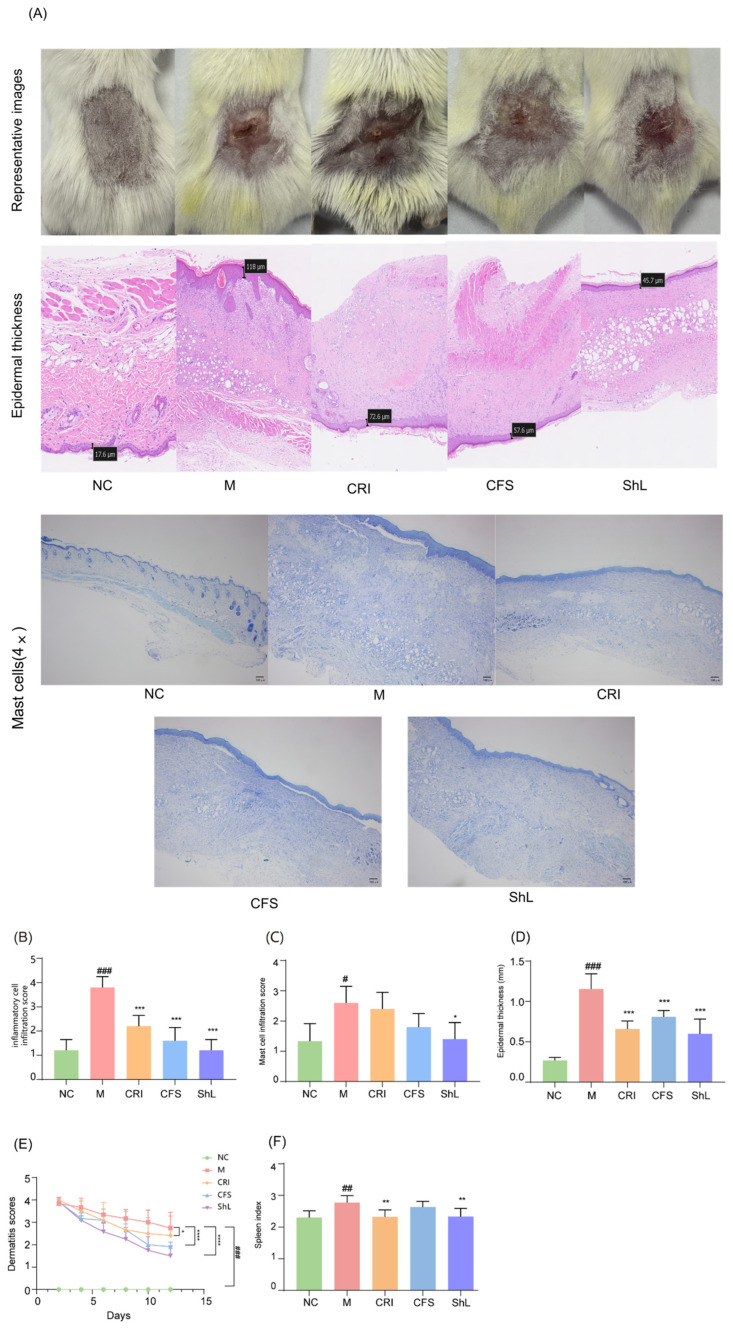
Topical CFS and ShL alleviate DNFB-induced AD-like dermatitis and reduce histopathological damage in mice. (**A**) Representative photographs of dorsal skin lesions and representative toluidine blue (TB) staining images showing dermal mast cells. (**B**) Semi-quantitative inflammatory cell infiltration score based on histological assessment. (**C**) Semi-quantitative mast cell infiltration score based on TB staining. (**D**) Epidermal thickness quantified from H&E-stained sections. (**E**) Clinical dermatitis score (sum of erythema/hemorrhage, dryness, erosion, and excoriation). (**F**) Spleen index (spleen weight/body weight). Data are presented as mean ± SD (*n* = 6 mice/group). * *p* < 0.05, ** *p* < 0.01, *** *p* < 0.001, **** *p* < 0.0001: compared with M group; # *p* < 0.05, ## *p* < 0.01, ### *p* < 0.001: compared with NC group.

Topical sensitization and challenge with DNFB successfully induced severe AD-like lesions in the dorsal skin of mice. As shown in the representative photographs (Figure 3A), the model group exhibited typical atopic features, including pronounced erythema, hemorrhage, edema, erosion, and skin dryness. In contrast, topical intervention with CFS, ShL, and the positive control (CRI) visibly mitigated these cutaneous symptoms. The dermatitis severity score was significantly elevated in the model group (*p* < 0.001). Both CFS and ShL treatments effectively reduced this score, with ShL exhibiting a pronounced recovery trend (Figure 3E).

Histopathological assessment via H&E staining further corroborated the clinical observations (Figure 3A). The model group displayed marked epidermal hyperplasia and massive infiltration of inflammatory cells into the dermis. Both CFS and ShL treatments significantly attenuated these pathological changes compared to the model group. Quantitatively, ShL and CFS treatment reduced epidermal thickness by 54.93% and 53.63%, respectively. Statistical analysis revealed that both treatments achieved comparable efficacy in reducing epidermal thickness and inflammatory cell infiltration scores, with no significant difference observed between the ShL and CFS groups (Figure 3B,D).

However, a critical divergence in efficacy was observed in specific immune markers. Toluidine blue (TB) staining was employed to visualize mast cell accumulation, a driver of allergic inflammation. While the model group showed a dense distribution of mast cells, only the ShL treatment resulted in a statistically significant reduction in the mast cell infiltration score compared to the model group (*p* < 0.01; Figure 3C). Notably, neither the CFS group nor the CRI group showed a significant difference from the model group in this specific parameter, highlighting a distinct capability of ShL to suppress mast cell accumulation in this model.

Furthermore, the evaluation of the spleen index—a marker of systemic immune activation—reinforced the superior immunomodulatory profile of ShL. The spleen index was significantly increased in the model group. Both ShL and the CRI treatments significantly reduced the spleen index compared to the model group (*p* < 0.01; Figure 3F), effectively restoring it towards normal levels. In contrast, CFS treatment failed to produce a significant reduction in the spleen index. These results collectively indicate that while both fractions effectively restore local skin structural integrity, the intracellular or cell-wall components present in ShL exhibit a stronger association with modulation of mast cell infiltration and selected systemic immune readouts in this model.

### 3.4. Modulation of Skin Cytokine Profiles and Barrier Gene Expression by CFS and ShL

**Figure 4:**
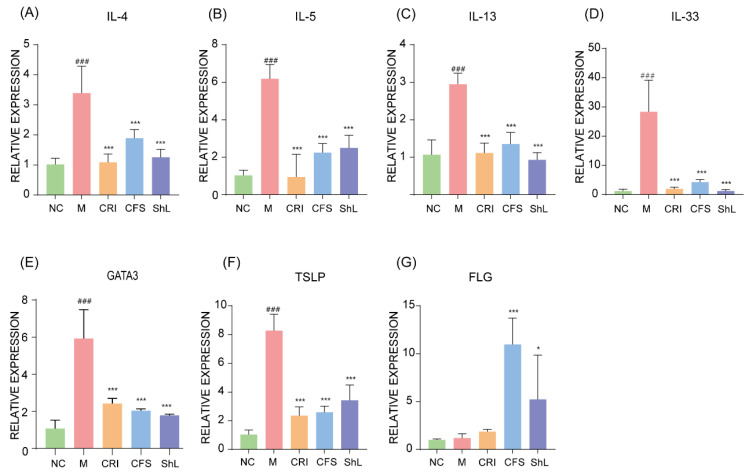
CFS and ShL downregulate type 2 inflammatory gene expression and increase filaggrin expression in lesional skin. RT-qPCR analysis of cytokine and barrier-related transcripts in dorsal skin: (**A**) IL-4, (**B**) IL-5, (**C**) IL-13, (**D**) IL-33, (**E**) GATA3, (**F**) TSLP, and (**G**) FLG. Data are presented as mean ± SD (*n* = 6 mice/group). * *p* < 0.05, *** *p* < 0.001: compared with M group; ### *p* < 0.001: compared with NC group.

To investigate the molecular mechanisms underlying the observed phenotypic improvements, we analyzed the expression profiles of key cytokines and barrier-related genes in skin lesions. The DNFB-induced model group exhibited a pronounced distinct type 2 immune bias, evidenced by significant upregulation of Th2-related cytokines (IL-4, IL-5, IL-13, IL-33; *p* < 0.001), the master transcription factor GATA3 (*p* < 0.01), and the epithelial alarmin TSLP (*p* < 0.001; Figure 4A–F). Treatment with both CFS and ShL effectively reversed these inflammatory signatures, significantly downregulating the mRNA expression of these pro-inflammatory mediators. In parallel, we assessed the integrity of the skin barrier by measuring filaggrin (FLG) expression. While FLG levels were severely suppressed in the model group, treatment with *S. harbinensis*-derived components restored its expression. Notably, CFS treatment induced a highly significant upregulation of FLG (*p* < 0.001; Figure 4G), while ShL treatment also resulted in a marked increase (*p* < 0.05; Figure 4G). These findings indicate that *S. harbinensis JNDM* components alleviate AD through concurrent suppression of type 2 inflammation and restoration of epidermal barrier integrity.

### 3.5. ShL Attenuates Systemic Immune Dysregulation

**Figure 5:**
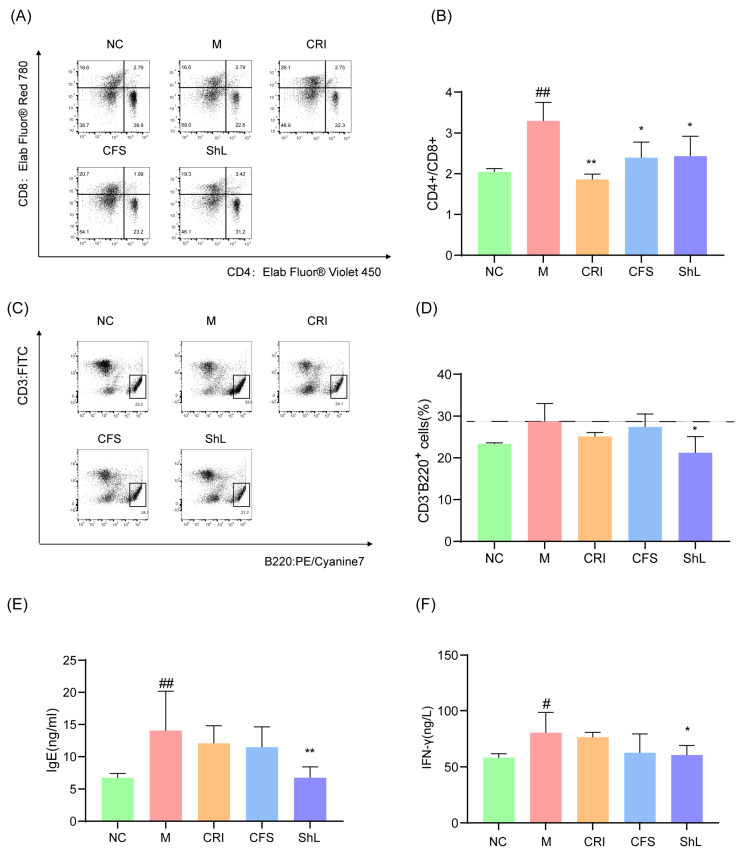
ShL improves systemic immune readouts in DNFB-induced AD mice. (**A**) Representative flow cytometry plots of splenic CD4^+^ and CD8^+^ T cells gated on CD3^+^ lymphocytes. (**B**) Quantification of the splenic CD4^+^/CD8^+^ T-cell ratio. (**C**) Representative flow cytometry plots of splenic B cells (B220^+^) gated on CD3^−^ cells. (**D**) Quantification of splenic B220^+^ B-cell frequency. (**E**) Serum total IgE measured by ELISA. (**F**) Serum IFN-γ measured by ELISA. Data are presented as mean ± SD (*n* = 6 mice/group). * *p* < 0.05, ** *p* < 0.01: compared with M group; # *p* < 0.05, ## *p* < 0.01: compared with NC group.

To comprehensively evaluate the impact of *S. harbinensis JNDM*-derived components on systemic immunity, we analyzed splenic lymphocyte populations and serum inflammatory markers. Flow cytometric analysis was employed to examine the distribution of T and B lymphocytes in the spleen. In the model group, the ratio of CD4^+^/CD8^+^ T cells was elevated compared to the normal control, indicating a helper T-cell-dominant immune imbalance (Figure 5A,B). Treatment with the CRI, CFS, and ShL all significantly reversed this imbalance. Specifically, CRI reduced the ratio with high significance (*p* < 0.01), while both CFS and ShL also produced statistically significant reductions (*p* < 0.05).

To evaluate the humoral immune response, we first analyzed the proportion of CD3^−^B220^+^ lymphocytes in the spleen. Although the model group exhibited a numerical increasing trend in B cell frequency compared to the normal control, and the ShL group showed a corresponding downward trend, these differences did not reach statistical significance among any of the experimental groups (*p* > 0.05; Figure 5C,D).

Given the stability of the B cell population, we further investigated whether the functional output of these cells was altered. We quantified serum biomarkers to assess the systemic immune state. The DNFB-induced model exhibited a complex inflammatory profile characteristic of the transition from acute to chronic AD. This was evidenced by a significant elevation in total IgE (*p* < 0.01; Figure 5E), a hallmark of Th2-driven sensitization, alongside a marked increase in IFN-γ (*p* < 0.05; Figure 5F), which signals the Th1 activation typical of chronic lesions [20]. Topical application of ShL effectively reversed this systemic dysregulation, yielding significant reductions in both serum IgE (*p* < 0.01) and IFN-γ (*p* < 0.05) compared to the model group. In stark contrast, neither the CFS nor the CRI groups achieved a statistically significant reduction in these serum markers.

In Figure 5F, serum IFN-γ levels in model group were significantly elevated compared to the NC group. Following topical intervention with positive drugs, CFS, and ShL, the abnormally elevated serum IFN-γ levels in the model group decreased to varying degrees. The downregulation effects in the CFS and ShL groups were comparable to those in the positive drug group. This finding exhibits clear pathological rationality, aligning with the core characteristic identified by Wasserer et al. in AD patients: “high IFN-γ subpopulations exhibit IFN-γ upregulation and coexist with Th2-type factors” [21]. This study confirms that AD is not a single immunological polarization but a heterogeneous disease dominated by Th2-type immunity accompanied by Th1-type immune activation. Elevated IFN-γ in the high IFN-γ subset reflects both the body’s antibacterial/antiviral defense response (e.g., significantly upregulated bacterial response pathways) and synergistically exacerbates keratinocyte apoptosis and chronic inflammation with factors like TNF-α, creating a dual “defense-damage” effect. In this study, the model group exhibited concurrent upregulation of cutaneous IL-4/IL-13 and elevated serum IFN-γ, precisely corroborating this “Th2-dominant, Th1-co-activated” mixed inflammatory pattern.

### 3.6. Topical Application of CFS and ShL Remodels Gut Microbiota and Boosts Butyrate Production

**Figure 6:**
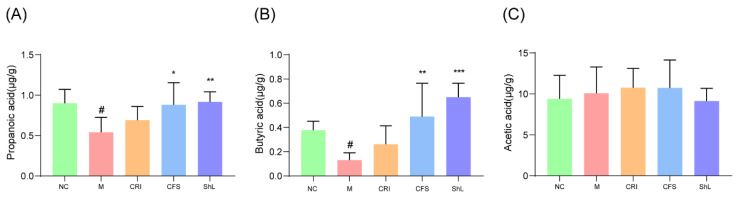
Topical postbiotics increase intestinal SCFA levels in DNFB-induced AD mice. Concentrations of (**A**) acetate, (**B**) propionate, and (**C**) butyrate measured by HPLC in fecal samples. Data are presented as mean ± SD (*n* = 6 mice/group). * *p* < 0.05, ** *p* < 0.01, *** *p* < 0.001: compared with M group; # *p* < 0.05 vs. NC group.

To investigate whether topical skin treatment could influence distal gut metabolism via the gut–skin axis, we analyzed fecal SCFA concentrations. The DNFB-induced AD model group exhibited significant dysregulation, characterized by significantly suppressed levels of propionate and butyrate compared to the NC group (*p* < 0.05; Figure 6A,B) with no significant change in the level of acetic acid (Figure 6C). Strikingly, topical intervention with both CFS and ShL effectively reversed these metabolic deficits. Both treatments restored fecal propionate concentrations to levels comparable to the NC group. Notably, butyrate levels were markedly elevated following treatment, exceeding NC levels, with ShL producing the strongest effect (*p* < 0.001). The butyrate concentration in the ShL group was approximately 4-fold higher than in the model group and 1.5-fold higher than in the NC group.

To determine whether the topical application of *S. harbinensis*-derived components influences the gut ecosystem, we analyzed the bacterial community structure using 16S rRNA gene sequencing.

**Figure 7:**
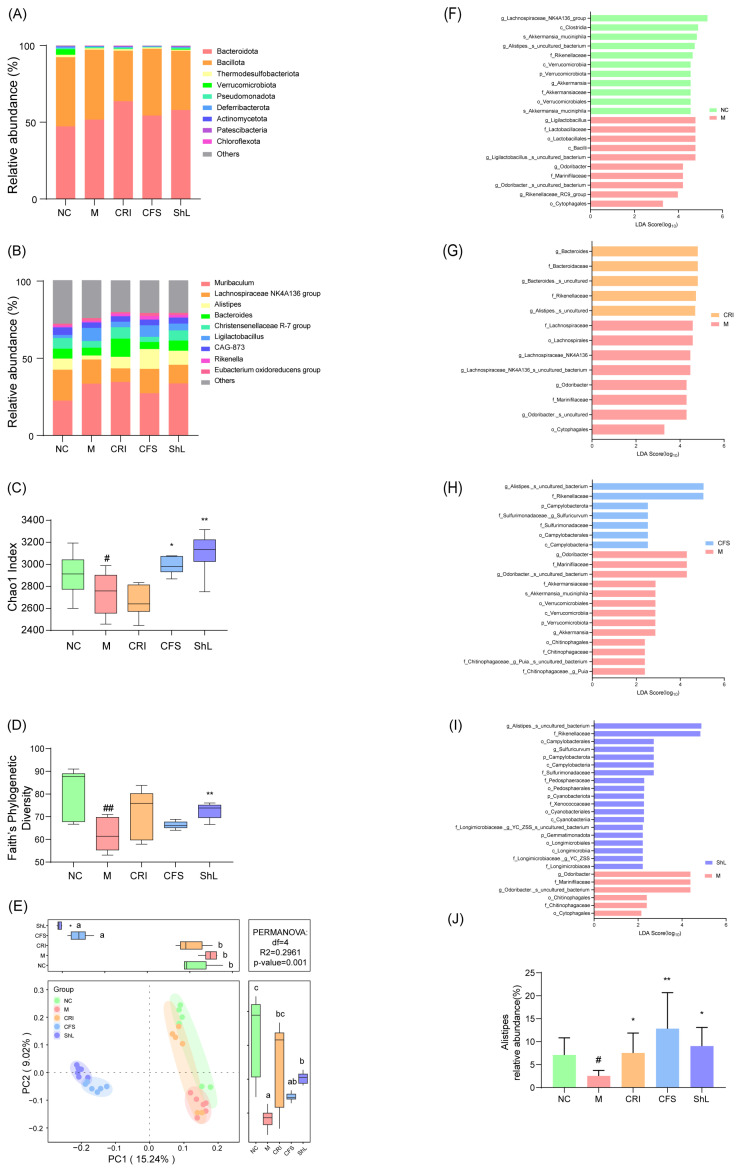
Topical CFS and ShL reshape gut microbiota composition and restore Alistipes abundance. (**A**) Relative abundance of bacterial taxa at the phylum level. (**B**) Relative abundance at the genus level. (**C**) Chao1 richness index. (**D**) Faith’s phylogenetic diversity index. (**E**) Principal coordinate analysis (PCoA) based on the Bray–Curtis β-diversity distances. PERMANOVA analysis indicated significant differences in microbial community composition among groups (R^2^ = 0.2961, *p* = 0.001). Pairwise PERMANOVA comparisons were additionally performed between the M group and each treatment group, and *p* values were adjusted using the Benjamini–Hochberg method. (**F**–**I**) LEfSe analysis identifying differentially abundant taxa (LDA score > 2.0, *p* ≤ 0.05) in pairwise comparisons between the Model group and (**F**) NC, (**G**) CRI, (**H**) CFS, and (**I**) ShL groups. (**J**) Relative abundance of the genus Alistipes. Data were expressed as mean ± SD (*n* = 6), * *p* < 0.05, ** *p* < 0.01: compared with M group; # *p* < 0.05, ## *p* < 0.01: compared with NC group. Different lowercase letters indicate statistically significant differences (*p* < 0.05). Groups sharing at least one common letter are not significantly different, while groups with no letters in common differ significantly.

First, we assessed alpha diversity to evaluate microbial richness and evenness. The DNFB-induced model group exhibited a significant contraction in gut microbial diversity, as evidenced by reduced Chao1 and Faith’s PD indices. Treatment with CFS and ShL effectively counteracted this dysbiosis, partially restoring the diversity indices towards healthy levels (Figure 7C,D). To visualize differences in the overall microbial community structure, Principal Coordinate Analysis (PCoA) was performed (Figure 7E). The first two principal coordinates explained 24.27% of the total variance (PCoA1: 15.24%; PCoA2: 9.03%). The PCoA plot revealed a striking clustering pattern that indicates a treatment-associated microbial shift. The NC, M and CRI groups clustered closely together, indicating that neither the disease state nor the Crisaborole Ointment treatment induced a global shift in the dominant microbial architecture sufficient to separate them in this analysis. In contrast, the CFS and ShL groups formed a tight, overlapping cluster that was distinctly segregated from the NC, Model, and CRI groups. This significant separation along the primary axis suggests that topical intervention with *S. harbinensis*-derived components—regardless of whether the supernatant or lysate was used—was associated with alterations in gut microbiota composition, accompanied by shifts in microbial community structure. This shift towards a distinct microbial profile correlated with increased fecal butyrate levels, suggesting treatment-associated microbiota compositional changes accompanied by altered SCFA output.

Taxonomic profiling at the phylum level revealed a consistent dominance of *Bacteroidota*, *Bacillota*, and *Thermodesulfobacteriota* across all groups. Collectively, these three phyla accounted for 93% to 99% of the total sequencing reads, forming a stable core microbiome structure (Figure 7A). At the genus level, the overall microbial community composition remained largely conserved across the experimental groups (Figure 7B). The six most abundant genera—*Muribaculum*, *Lachnospiraceae NK4A136 group*, *Alistipes*, *Bacteroides*, *Christensenellaceae R-7 group*, and *Ligilactobacillus*—constituted approximately 64.53% to 73.09% of the total microbiota. Among these high-abundance genera, significant variations were observed specifically in the relative abundance of *Alistipes*. In the model group, the proportion of *Alistipes* was significantly lower than that of the Normal Control group (*p* < 0.05). However, intervention with CFS, ShL, and the CRI significantly reversed this reduction, resulting in a marked increase in *Alistipes* abundance compared to the model group (*p* < 0.05; Figure 7I).

To identify specific bacterial taxa distinguishing the gut microbiota of the experimental groups, we performed Linear Discriminant Analysis Effect Size (LEfSe) analysis via pairwise comparisons between the M group and each of the other groups. Taxa with an LDA score (log10) ≥ 2.0 were considered statistically significant biomarkers (Figure 7F–J). The NC group was characterized by a significant enrichment of the *Lachnospiraceae NK4A136 group*, *Alistipes*, and *Akkermansia muciniphila*. The *Lachnospiraceae NK4A136 group* is established as a primary butyrate acid in the healthy gut [22], while *Akkermansia muciniphila* is renowned for enhancing mucosal barrier integrity and preventing the translocation of bacterial endotoxins (LPS) into the systemic circulation [23]. In the M group, the microbial landscape shifted significantly. This group was dominated by *Ligilactobacillus*, a major lactate producer, and *Odoribacter*. Notably, the emergence of *Odoribacter*—also a butyrate-producing genus [24]—suggests a compensatory shift where it replaced the *Lachnospiraceae NK4A136 group* as the dominant butyrate acid in the disease state, potentially reflecting an altered metabolic network under inflammatory stress. Crucially, intervention with CFS, ShL and the CRI specifically enriched bacteria from the family *Rikenellaceae*, particularly the genus *Alistipes*. The CRI group was further distinguished by the specific enrichment of *Bacteroides* alongside *Alistipes*. *Alistipes*, a member of the phylum *Bacteroidota*, has been associated with anti-inflammatory and SCFA-related functions in several contexts, although its role appears to be context-dependent across disease settings [25,26]. Mechanistically, *Alistipes* is capable of fermenting undigested carbohydrates to produce propionate. This aligns with our SCFA findings mentioned above and suggests that the restoration of *Alistipes* abundance coincided with the partial recovery of intestinal propionate levels, which in turn regulates T-cell differentiation and attenuates cutaneous inflammation [27]. Because the microbiome analysis was based on relative abundance profiling, these findings should be interpreted as treatment-associated compositional shifts rather than definitive evidence of microbiome-wide functional reprogramming. A complementary MaAsLin3 analysis was also performed and showed only partial overlap with the LEfSe-identified taxa, indicating method-dependent variability in differential abundance results.

### 3.7. Correlation Analysis of Gut Microbiota with Skin Barrier and Immune Indices

**Figure 8:**
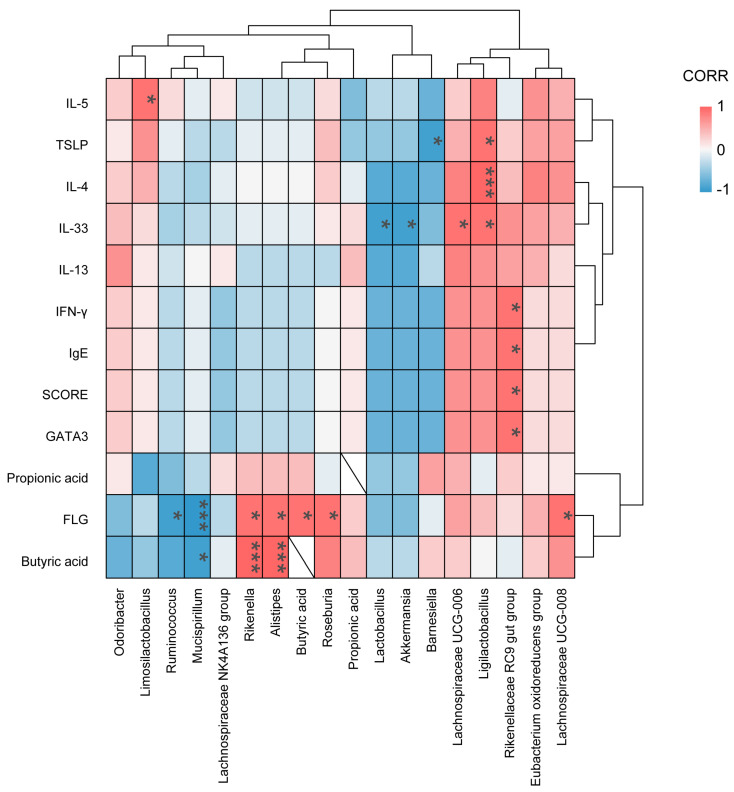
Correlation analysis between gut microbiota, SCFAs, immune markers, and skin barrier indicators. Spearman correlation coefficients are shown as a heatmap. Positive correlations are indicated in red and negative correlations in blue. Statistical significance was determined using the Spearman correlation with the Benjamini–Hochberg false discovery rate (FDR) correction. * FDR-adjusted * *p* < 0.05, *** *p* < 0.001.

To explore potential associations between specific gut microbial taxa and the phenotypic features of AD, Spearman’s rank correlation analysis was performed between key bacterial genera and clinical indices, skin barrier markers, and cytokine profiles (Figure 8).

A distinct cluster of positive correlations was observed among SCFA-producing bacteria, metabolic output, and skin barrier markers. *Alistipes* and *Rikenella* exhibited a strong positive correlation with fecal butyrate levels (*p* < 0.001), consistent with their known metabolic capabilities [28,29,30]. Notably, these taxa, along with other known butyrate producers such as *Roseburia* and *Lachnospiraceae UCG-008*, showed significant positive associations with the skin barrier protein FLG (*p* < 0.05). Conversely, *Mucispirillum* was negatively associated with butyrate (*p* < 0.05) and FLG (*p* < 0.001), while *Ruminococcus* was also showed a negative correlation with FLG (*p* < 0.05). These statistical associations suggest the abundance of specific butyrate-producing genera may be associated with improved skin barrier integrity.

## 4. Discussion

Atopic dermatitis is a complex dermatological syndrome driven by the interplay of epidermal barrier disruption, immune dysregulation, and microbial dysbiosis. While topical corticosteroids and calcineurin inhibitors remain the standard of care, their long-term use is often constrained by adverse effects, necessitating the development of safer biological alternatives [31]. In this study, we provide comprehensive evidence that the topical application of *Schleiferilactobacillus harbinensis JNDM*-derived postbiotics—specifically the lysate (ShL) and cell-free supernatant (CFS)—attenuates DNFB-induced AD-like symptoms in mice. Our findings indicate a multi-layered pattern of response involving restoration of the skin barrier, modulation of systemic immune readouts, and concurrent changes in gut microbiota composition potentially related to gut–skin axis pathway.

The integrity of the stratum corneum is paramount in preventing allergen penetration [32]. In vitro, ShP was used as a crude whole-postbiotic preparation for initial bioactivity screening. ShP significantly upregulated the expression of key epidermal barrier proteins, including filaggrin, loricrin, and involucrin in HaCaT keratinocytes and accelerated wound closure [33,34]. This direct regenerative effect was translated in vivo, where both CFS and ShL treatments significantly reduced epidermal thickness and restored FLG expression in dorsal skin lesions. Concurrently, we observed a potent anti-inflammatory effect. In LPS-stimulated macrophages, ShP dose-dependently inhibited NO production and suppressed iNOS and COX-2 expression, suggesting blockade of the NF-κB or MAPK inflammatory cascades [35]. In the AD mouse model, this manifested as a considerable downregulation of Th2-associated cytokines and the alarmins TSLP. The simultaneous reduction in elevated serum IFN-γ indicates that these postbiotics do not merely suppress Th2 immunity but rather re-equilibrate the Th1/Th2 balance, addressing the heterogeneous “mixed-type” inflammation often seen in chronic AD lesions [21].

A critical finding of this study is the differential efficacy between the ShL and the CFS. While both fractions effectively improved local skin symptoms, ShL exhibited superior systemic immunomodulatory properties. Only ShL significantly reduced the spleen index and effectively suppressed mast cell infiltration in the dermis. These findings suggest that intracellular or cell-wall-associated components present in the lysate may contribute to differential host responses compared with soluble extracellular components [36].

Our flow cytometric and serological analyses revealed a nuanced mechanism of systemic immune regulation. Treatment with ShL significantly reduced serum IgE levels (*p* < 0.01), a key driver of atopic sensitization. However, contrary to the expectation of B-cell depletion, we observed no statistically significant alteration in the total proportion of splenic B cells. This decoupling of cell abundance from functional output implies that ShL acts as a functional modulator rather than a cytotoxic agent. It likely operates by intercepting the downstream signaling required for class-switch recombination to IgE or by inhibiting plasma cell differentiation, rather than by broadly depleting the B-cell pool [37,38]. This mechanism offers a distinct safety advantage, as it dampens the allergic drive without compromising the host’s general humoral immunity. Meanwhile, because crisaborole primarily acts locally through PDE4 inhibition, differences in systemic immune markers between treatment groups should be interpreted cautiously and may reflect differences in mechanism of action rather than overall therapeutic superiority.

Perhaps the most intriguing finding is that topical skin intervention induced considerable changes in the distal gut metabolome. AD mice exhibited a depletion of propionate and butyrate, consistent with clinical observations in atopic patients [39]. Strikingly, the ShL group exhibited a 4-fold elevation in fecal butyrate concentration compared to the model group, surpassing even the baseline levels of the NC group (approximately 1.5-fold higher). Butyrate has been reported to support epithelial barrier integrity and immunoregulation in other experimental systems, although these downstream host pathways were not directly examined in the present study [40]. The restoration of these metabolites suggests that signals from the ameliorated skin environment (possibly via reduced systemic circulation of inflammatory cytokines like IL-4 and IL-13) relieve the stress on the gut ecosystem, allowing beneficial metabolic networks to recover.

This metabolic surge aligns with our 16S rRNA sequencing data. PCoA analysis revealed that CFS and ShL treatments did not merely revert the microbiome to the “Normal” state but shifted it toward a unique structural configuration. Taxonomic profiling identified *Alistipes* as a key responder [41]. The abundance of *Alistipes* was depleted in the model group but significantly restored by therapeutic intervention. LEfSe and correlation analyses further substantiated a “beneficial axis”: the abundance of *Alistipes* was strongly positively correlated with fecal butyrate levels (*p* < 0.001) and skin FLG expression (*p* < 0.05). Conversely, the disease state was characterized by a “compensatory shift” where *Odoribacter* and *Ligilactobacillus* dominated, correlating with elevated Th2 cytokines (IL-4, IL-33) [24,42,43]. While we cannot infer strict causality from 16S data, these statistical associations support the hypothesis that ShL treatment was associated with a gut microbial configuration enriched in *Alistipes* and other SCFA-related taxa, coinciding with increased propionate and butyrate levels. These metabolites may represent one possible link between gut microbial alterations and systemic or skin-associated effects, although this putative gut–skin axis was not directly validated in the present study [13,44,45].

Despite these encouraging findings, several limitations should be acknowledged. First, although topical postbiotic treatment was associated with concurrent changes in gut microbiota composition and fecal SCFA output, the present study does not establish a direct causal skin–gut signaling pathway. Additional studies such as tracer-based tracking, intestinal permeability assays, immune-cell trafficking analysis, or microbiota-transfer approaches would be required to define the mechanistic route more rigorously. Microbiome functional changes were inferred from taxonomic profiles and SCFA quantification rather than directly validated by metagenomic or metabolomic analyses. Therefore, conclusions regarding metabolic modulation should be interpreted cautiously. Second, although *Alistipes* abundance and butyrate levels emerged as prominent correlates of treatment response, their specific causal contribution remains unresolved. Third, the in vitro assays were performed using ShP, whereas the in vivo study compared CFS and ShL. This design was intended to combine preliminary bioactivity screening with fraction-specific in vivo evaluation, but it does not establish a strict one-to-one mechanistic correspondence across experimental platforms. Finally, although the revised study now includes comparative physicochemical characterization of CFS and ShL, including SDS-PAGE profiling and particle property analysis (particle size, PDI, and zeta potential), deeper molecular identification of the active bioactive components will require future proteomic, metabolomic, or targeted fractionation studies.

## 5. Conclusions

Our study demonstrates that the topical application of *S. harbinensis JNDM*-derived CFS and ShL effectively alleviates DNFB-induced AD in mice through a multi-targeted response involving the restoration of epidermal barrier integrity, the modulation of immune readouts, and concurrent changes in gut microbial composition and fecal SCFA output, suggesting the possible involvement of gut–skin axis-related pathways. ShL exhibits superior efficacy in modulating allergic inflammation and systemic immunity compared to CFS, making it a more promising postbiotic candidate for AD treatment. Future studies should focus on identifying the key bioactive components of *S. harbinensis* postbiotics, optimizing their formulation, and conducting clinical trials to validate their efficacy and safety in human AD patients, which will accelerate the translation of probiotic-based postbiotics into clinical practice for AD treatment.

## Figures and Tables

**Table 1:** Primers used for RT-qPCR in HaCaT cells.

	Forward Primer (5′–3′)	Reverse Primer (5′–3′)
GAPDH	GGAGCGAGATCCCTCCAAAAT	GGCTGTTGTCATACTTCTCATGG
IL-1β	AAACAGATGAAGGTGCTCCTT	TGGAGAACACCACTTGTTGC
IL-6	AAATTCGGTACATCCTCGACGGCA	AGTGCCTCTTTGCTGCTTTCACAC
IL-8	AAGCTGGCCGTGGCTCTCTTG	AGCCCTCTTCAAAAACTTCTC
CCL17	CTTCTCTGCAGCACATCC	AAGACCTCTCAAGGCTTTG
CCL22	AGGACAGAGCATGGATCGCCTACAGA	AATGGCAGGGAGGTAGGGCTCCTGA
FLG	TGGAGATGGCACAGTAAAGGAG	GGCTTCTTCCTTATTGCTGCTC
INV	GCTCAGATGCTGAAGGAGAAC	GGCTTCTTCCCTTCACCTTC
LOR	CAGCAACAAGATCCCTGAGC	ATGGCTTCCAGTTGCAGTAGG

**Table 2:** Primers used for RT-qPCR in RAW264.7 cells.

	Forward Primer (5′–3′)	Reverse Primer (5′–3′)
GAPDH	GGAGCGAGATCCCTCCAAAAT	GGCTGTTGTCATACTTCTCATGG
IL-1β	AAACAGATGAAGGTGCTCCTT	TGGAGAACACCACTTGTTGC
IL-6	AAATTCGGTACATCCTCGACGGCA	AGTGCCTCTTTGCTGCTTTCACAC
TNF-α	AAGCTGGCCGTGGCTCTCTTG	AGCCCTCTTCAAAAACTTCTC
iNOS	CTTCTCTGCAGCACATCC	AAGACCTCTCAAGGCTTTG
COX2	AGGACAGAGCATGGATCGCCTACAGA	AATGGCAGGGAGGTAGGGCTCCTGA

**Table 3:** Primers used for RT-qPCR in mouse dorsal skin.

	Forward Primer (5′–3′)	Reverse Primer (5′–3′)
GAPDH	AGGTCGGTGTGAACGGATTTG	GGGGTCGTTGATGGCAACA
IL-4	GGTCTCAACCCCCAGCTAGT	GCCGATGATCTCTCTCAAGTGAT
IL-5	TGCAGACGATGAGGCTTCCT	GAGAATCACTTGAACCCAGGAGC
IL-13	CCTGGCTCTTGCTTGCCTT	GGTCTTGTGTGATGTTGCTCA
IL-33	TCCAACTCCAAGATTTCCCCG	CATGCAGTAGACATGGCAGAA
GATA3	GGGGCCTCTGTCCGTTTAC	TCCAGCTTCATGCTATCTGGC
FLG	GATCTGGTTCCACCGAAAGA	TGTAGCTTGCCTCCCAGTCT
TSLP	ACGGATGGGGCTAACTTACAA	CAGAGTCAAATTGGAGTTTGTG

## Data Availability

The 16S rRNA sequencing data generated in this study have been deposited in the BIG Data Center (https://bigd.big.ac.cn/) under accession number CRA040514 (23 August 2028). Other datasets supporting the findings of this study are available from the corresponding author upon reasonable request.

## Supplementary Materials

The following supporting information can be downloaded at https://www.mdpi.com/article/10.3390/microorganisms14040913/s1. Table S1: Physicochemical characterization of CFS and ShL preparations. Figure S1: SDS-PAGE profiles of ShL and CFS preparations. Figure S2: Representative sequential gating strategy for splenic lymphocyte subset analysis. Figure S3: Complementary differential abundance analysis using MaAsLin3.
