# Supplementary material for: Schleiferilactobacillus harbinensis JNDM Postbiotics Alleviate Atopic Dermatitis with Concurrent Changes in Gut Microbiota and Fecal SCFAs
Source: Microorganisms. 2026 Apr 17;14(4):913. doi: 10.3390/microorganisms14040913 (PMC13118722; doi:10.3390/microorganisms14040913)
Supplement: Supplementary file 1 [file microorganisms-14-00913-s001.zip › Supplementary Materials.pdf]

Supplementary Table S1. Physicochemical characterization of CFS and ShL preparations

	CFS	ShL
Total sugar(mg/L)	7.68±0.4	0.67±0.01
Total protein(μg/mL)	252.46±17.00	23.51±1.15
pH	3.67±0.04	7.34±0.01
Total acidity (expressed as lactic acid, g/L)	18.42	
PDI		0.30±0.04
zeta potential(mV)		-14.73±0.97

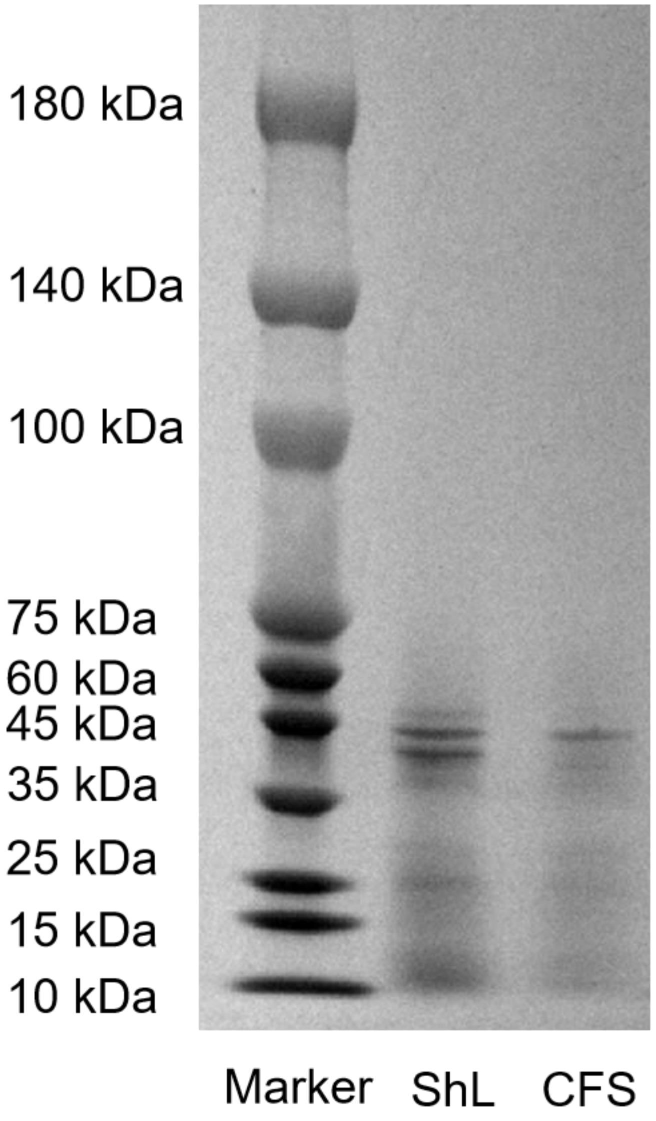

Supplementary Figure S1. SDS-PAGE profiles of ShL and CFS preparations.

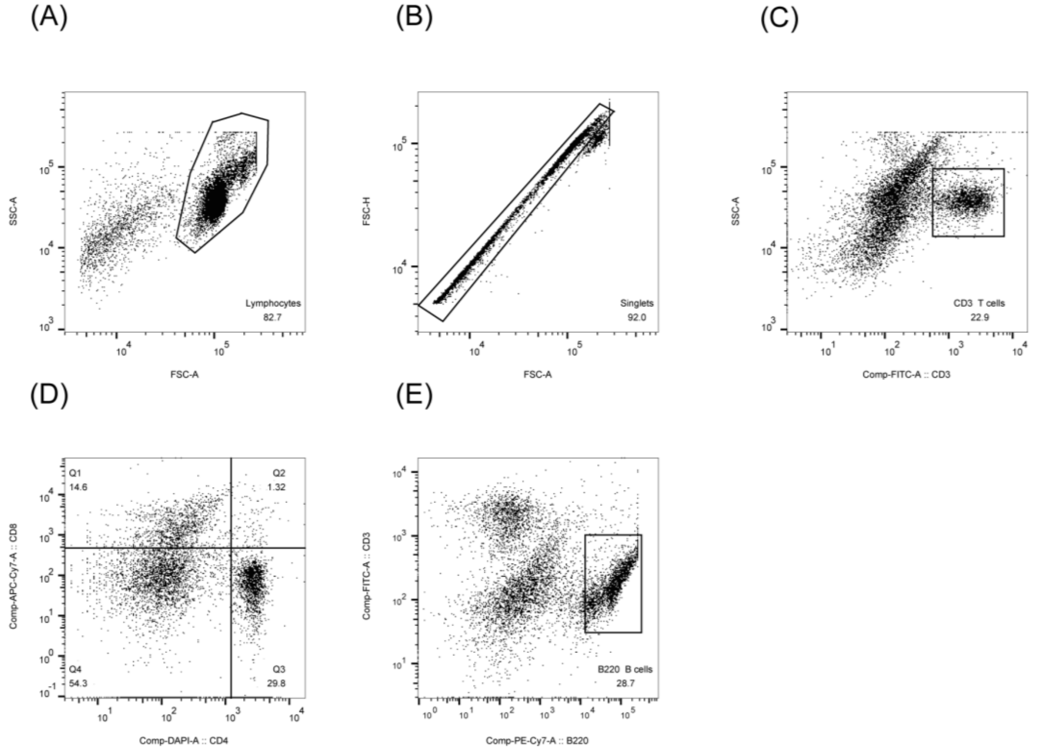

Supplementary Figure S2. Representative sequential gating strategy for splenic lymphocyte subset analysis. (A) Lymphocytes were identified using FSC-A versus SSC-A to exclude debris.(B) Singlets were selected using FSC-H versus FSC-A to exclude doublets. (C) $CD3^+$ T cells were gated within the singlet lymphocyte population. (D) $CD4^+$ helper T cells and $CD8^+$ cytotoxic T cells were identified within $CD3^+$ cells. (E) $B220^+$ B cells were identified within the $CD3^-$ population.

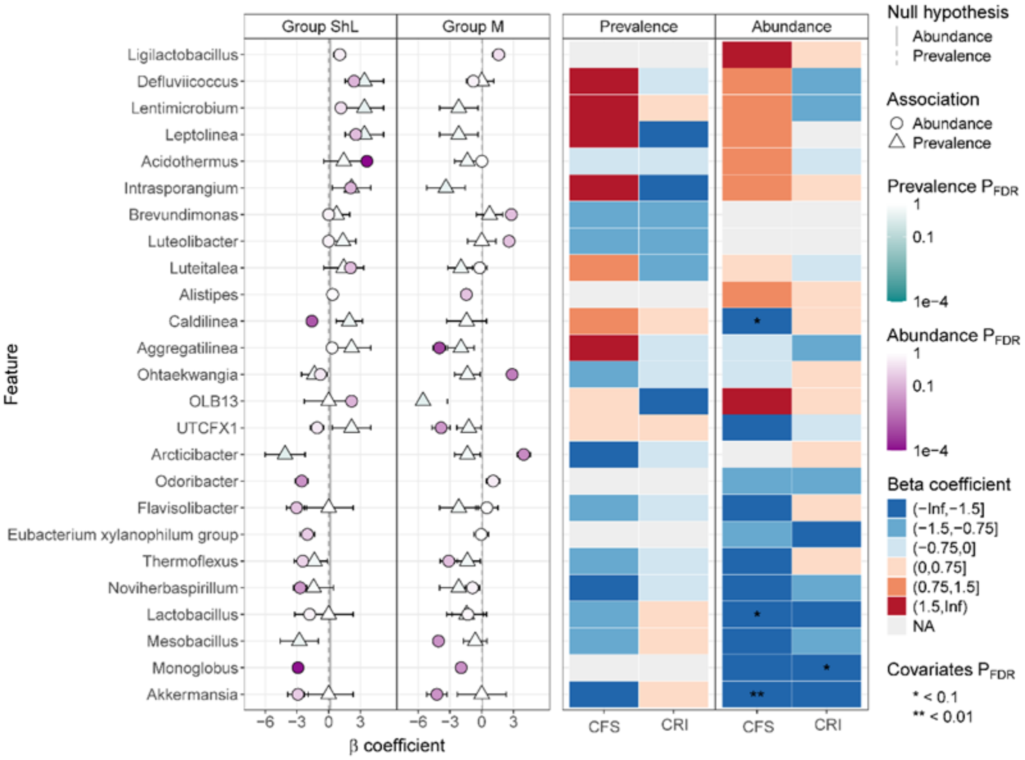

Supplementary Figure S3. Complementary differential abundance analysis using MaAsLin3. Results are presented as a sensitivity analysis to assess the robustness of taxa identified by LEfSe.
